# An Effective Multi-Stage Liposomal DNA Origami Nanosystem for In Vivo Cancer Therapy

**DOI:** 10.3390/cancers11121997

**Published:** 2019-12-12

**Authors:** Stefano Palazzolo, Mohamad Hadla, Concetta Russo Spena, Isabella Caligiuri, Rossella Rotondo, Muhammad Adeel, Vinit Kumar, Giuseppe Corona, Vincenzo Canzonieri, Giuseppe Toffoli, Flavio Rizzolio

**Affiliations:** 1 Pathology unit, Centro di Riferimento Oncologico di Aviano (CRO) IRCCS, Aviano 33081, Italy; stpalazz85@gmail.com (S.P.); concettarussospena@gmail.com (C.R.S.); icaligiuri82@gmail.com (I.C.); rossellaross1988@gmail.com (R.R.); addistar60@yahoo.com (M.A.); vcanzonieri@cro.it (V.C.); 2 Clinical and Experimental Pharmacology unit, Centro di Riferimento Oncologico di Aviano (CRO) IRCCS, Aviano 33081, Italy; m_hadla@hotmail.com (M.H.); vinitiitr@gmail.com (V.K.); gtoffoli@cro.it (G.T.); 3 Department of Molecular Sciences and Nanosystems, Ca’ Foscari University of Venice, Venice 30172, Italy; 4 Amity Institute of Molecular Medicine & Stem Cell Research, Amity University, Noida 201313, India; 5 Immunopathology and Cancer Biomarkers unit, Centro di Riferimento Oncologico di Aviano (CRO) IRCCS, Aviano 33081, Italy; giuseppe.corona@cro.it; 6 Department of Medical, Surgical and Health Science, University of Trieste, Trieste 34137, Italy

**Keywords:** DNA origami, liposome, breast cancer, remote loading, doxorubicin, acute toxicity, organoids

## Abstract

DNA origami systems could be important candidates for clinical applications. Unfortunately, their intrinsic properties such as the activation of non-specific immune system responses leading to inflammation, instability in physiological solutions, and a short in vivo lifetime are the major challenges for real world applications. A compact short tube DNA origami (STDO) of 30 nm in length and 10 nm in width was designed to fit inside the core of a stealth liposome (LSTDO) of about 150 nm to remote load doxorubicin. Biocompatibility was tested in three-dimensional (3D) organoid cultures and in vivo. Efficacy was evaluated in different cell lines and in a xenograft breast cancer mouse model. As described in a previous work, LSTDO is highly stable and biocompatible, escaping the recognition of the immune system. Here we show that LSTDO have an increased toleration in mouse liver organoids used as an ex vivo model that recapitulate the tissue of origin. This innovative drug delivery system (DDS) improves the antitumoral efficacy and biodistribution of doxorubicin in tumor-bearing mice and decreases bone marrow toxicity. Our application is an attractive system for the remote loading of other drugs able to interact with DNA for the preparation of liposomal formulations.

## 1. Introduction

Cancer is the second major cause of death worldwide every year [1]. The standard methods to treat this deadly disease are surgery and chemotherapy with cytotoxic antitumor drugs [2]. Chemotherapeutic drugs possess unspecific targeting with a large biodistribution, leading to several side effects that make chemotherapy painful and even fail in some cases [3]. To overcome these limitations, the use of nanotechnologies marked important progressions in the development of a drug delivery system (DDS) able to improve the chemical, physical and pharmacological properties of drugs [4]. Ideally, the DDS to be applied in clinic should have the following properties: low toxicity, the ability to cross physiological barriers, high stability in body fluids (in particular in the blood stream), high loading efficiency and controlled drug release [5]. In order to meet the clinical requirements, nanotechnology plays a vital role in the development of smart delivery systems with excellent features such as size (around 100 nm), various functionalization and a high surface to volume ratio of nanomaterials. Recently, even though different nanomaterials like polymers and metal nanoparticles have been proposed for the development of nanostructures for smart DDS [6], the construction of biocompatible and stable vehicles for in vivo applications still remains the challenge for clinical therapy [7]. In the last decade, DNA technology received considerable attention for many attractive features such as easy synthesis in the predicted shape, precise nanopatterning, and mechanical rigidity, making DNA an interesting candidate for biomedical applications [8]. In the last three decades, scientists have developed different shapes of DNA nanostructures but, since 2000, the field was innovated with the advent of a folding technique called DNA origami [3]. DNA origami provides a platform for next generation DDS, becoming a potential candidate for clinics. In this regard, Hogberg et al. tested two different kinds of DNA origami structures for doxorubicin loading on three different breast cancer cell lines [5]. Ding et al. used self-assembled DNA origami as a carrier for doxorubicin that was able to circumvent drug resistance on multi-drug resistant breast cancer cells (MDR-MCF7) [6]. Yang et al. designed and synthesized triangular shaped DNA origami for the loading of doxorubicin to be used as a vehicle for in vivo cancer therapy [7]. Even though DNA nanostructures are an interesting DDS, they possess some limitations which prevent their in vivo application [9]. In particular, DNA nanovehicles, once in the blood stream, are rapidly recognized by circulating DNAses and by the immune system, inducing inflammatory responses. The possibility to encapsulate DNA nanostructures in double-layer membranes or in a protein coating could avoid DNAse degradation and immune system activation, improving pharmacokinetics, bioavailability and biodistribution [10,11,12].

Among the clinical-grade nanoparticle-based technologies, liposomal technology has become a very successful and rapidly developing area of preclinical and clinical research. With the advantages of biocompatibility, biodegradability, low toxicity, and aptitude to trap both hydrophilic and lipophilic drugs [13] as well as a desirable accumulation in tumor tissues [14], liposomes are very attractive and have been extensively investigated as a DDS. The size of liposomes makes them suitable to cross the fenestrations of blood vessels in the tumor site and to remain entrapped in the extracellular matrix. The long circulation time in the blood stream is obtained by adding polyethylene glycol (PEG) chains on the surface of the liposome, which allow it to escape the reticuloendothelial system (RES) and the immune system [15]. On this topic, Doxil represents one of the main examples of a liposomal formulation of doxorubicin. This anthracycline is loaded inside the liposome through a process called remote loading, by which doxorubicin precipitates inside the liposome, avoiding its release [16]. The net result is a high drug/lipid ratio that is essential for clinical application. Unfortunately, the liposomal system has certain limitations such as reaching an efficient drug loading only with weak base or acid molecules. Therefore, to overcome these limitations and improve the DNA nanostructure limitations (biodegradation and immune system activation), our group developed, for the first time, a robust bullet biomimetic system by making short tube DNA origami (STDO) of approximately 30 nm in length and 10 nm in width with high stability in physiological conditions for up to 48 h [17]. The compact size of STDO precisely fittted inside a stealthy liposome of about 150 nm and doxorubicin was efficiently remote loaded in liposomes (LSTDO). The LSTDO system had a controlled release at pH 7.4 with an increased release rate in acidic conditions (pH 5.5) typical of the tumor microenvironment. LSTDO also improved the biocompatibility of DNA origami injected into immunocompetent mice (FVB/N) [17]. Therefore, there was a need to test the system (LSTDO) in vivo in order to determine if it could be used in clinics for a better cancer therapy.

**Figure 1:**
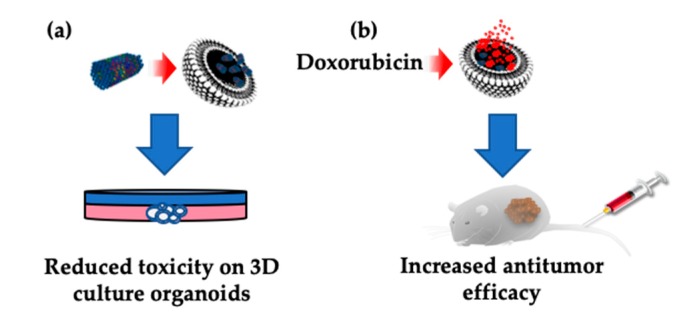
Schematic representation of the new liposomal/origami technology for the drug delivery of doxorubicin. (**a**) Liposomal short tube DNA origami (LSTDO) decreases the adverse effect of STDO on mouse liver derived organoids. (**b**) LSTDO-doxo increases the antitumor effect of doxorubicin on mice bearing breast cancer tumor cells.

In this work, we studied the LSTDO remotely loaded with doxorubicin for in vivo applications. The combined properties of liposomes and DNA origami allowed for the introduction of a biocompatible innovative system for the delivery of doxorubicin, which improved tumor accumulation of the drug and efficiently inhibited tumor growth in mice (Figure 1).

## 2. Results

### 2.1. Synthesis and Characterization 

The original data were published by our group in Palazzolo et al. [17]. Briefly, STDO was assembled in a one-step reaction. The expected dimensions were about 30 nm in length and 10 nm in width. After synthesis, STDO was purified by PEG precipitation as described by Stahl et al. [18]. The lipid composition used to create the liposomes to encapsulate the STDO was derived from Doxil. Liposomes were synthesized by applying the membrane extrusion method in a physiological buffer containing STDO (see Materials and Methods). After this process LSTDO were purified by removing excess of STDO with a cationic resin able to remove free DNA origami without interfering with liposomes. The DDS were analyzed by transmission electron microscopy (TEM) and dynamic light scattering (DLS). The calculated hydrodynamic radius of the STDO and of the LSTDO before and after purification from unencapsulated STDO were 37.6 ± 5.4 nm, 170.1 ± 67.0 nm and 163.9 ± 54.2 nm, respectively (Supplementary Materials Figure S1).

LSTDO was able to load doxo at about 50% (w/w) loading efficiency. The release of LSTDO was pH dependent and increased at pH 5.5, a typical condition of the tumor microenvironment. Other data, including STDO agarose gel electrophoresis, size distribution and stability in physiological conditions were described in Palazzolo et al. [17].

### 2.2. Liver Organoid Toxicity

**Figure 2:**
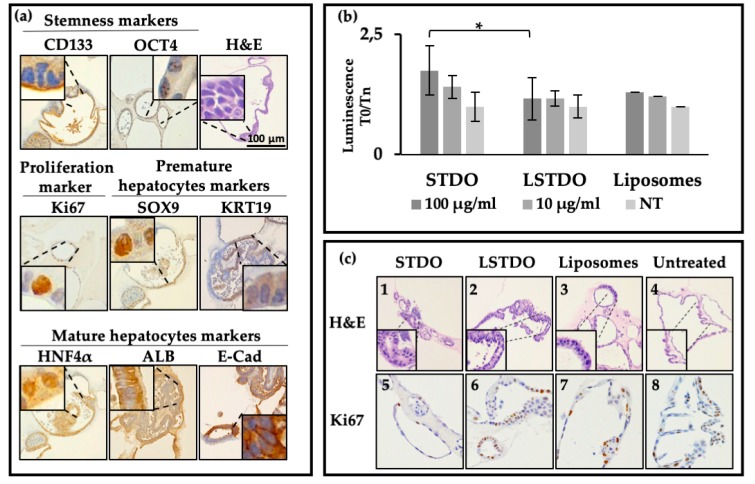
DNA origami-induced toxicity on mouse liver organoids. (**a**) Cytomorphological and immunohistochemical characterization of mouse liver organoids as a model to test the toxicity induced by origami. (**b**) Induced apoptosis (Cas 3/7 assay) as a toxicity marker of LSTDO and STDO. (**c**) Cytomorphological changes (H&E) induced by STDO (Panel 1) and LSTDO (Panel 2) compared with the control and liposomes (Panel 3 and 4, respectively). Ki67 expression in mouse liver organoids treated with STDO (Panel 5) and LSTDO (Panel 6) compared with the control and liposomes (Panel 7 and 8, respectively). Image magnification: 20×. (* *p*-value < 0.05).

Mouse liver organoids were isolated from eight weeks-old C57BL6 mice. Organoids were phenotypically characterized by histological and immunohistochemical (IHC) staining for the most common liver markers (Figure 2). In accordance with cyto-morphological evidence reported in the literature [19], microscopic examination of haematoxylin and eosin (H&E) staining showed that liver organoids had the typical features of hepatic progenitors and mature hepatocytes. Liver organoids retained their stemness and proliferative potential, confirmed respectively by the strong immunopositivity for CD133 and octamer-binding transcription factor 4 (OCT4) stem cell markers and Ki67 proliferation marker. Transcription factor SOX9 and cytokeratin 19 (KRT19)-immunopositive cells, typical markers of premature ductal hepatocytes, were observed in the single-layer epithelium (Figure 2a). Cells of the stratified epithelium differentiated into mature hepatocytes and resulted in albumin (ALB+) and HNF4α-positive (HNF4α+) cells (Figure 2a). Therefore, our results highlight that mouse liver organoids maintain their commitment to their tissue of origin.

On this note, a single-layer epithelium was composed of cuboidal cells positive for E-cadherin (E-Cad+) (Figure 2a), which surround a central lumen and alternated with pseudostratified epithelium in a structure that resembles a primordial hepatic diverticulum.

Since the liver is the organ which accumulates the majority of compounds administered intravenously and is the organ which detoxifies the blood from toxic substances, we decided to test if our DDS is biocompatible with liver organoids to predict in vivo biocompatibility. Organoids were plated and treated with STDO, LSTDO and liposomes at two different concentrations (100 µg/mL and 10 µg/mL). After 24 h, the caspase 3/7 assay was used to determine the activation of apoptotic enzymes (Figure 2b). The level of caspase 3/7 was increased significantly after treatment of liver organoids with 100 µg/mL of STDO compared with organoids treated with LSTDO or only liposomes. The quantity used is the same amount employed to treat mice.

Morphological examination of H&E staining revealed that organoids treated for 48 h with 100 µg/mL STDO exhibit cytostructural changes. Indeed, compared with the control, STDO induced a loss of epithelial cells and a loss of organoid structural complexity. Moreover, cellular atypia and nuclear karyorrhexis and karyolysis, accompanied by irregular clumping of chromatinic material, were detectable (Figure 2c, Panel 1). Treatment with 100 µg/mL of LSTDO did not affect the general structure of organoids, displaying unremarkable changes such as slight visible nuclear pyknosis and slight cellular atypia (Figure 2c, Panel 2). Untreated and liposome-treated mouse liver organoids appeared as similar glandular structures with active proliferations (Figure 2c, Panels 3 and 4).

In order to assess if STDO-induced cytotoxicity could affect organoid cell proliferation, immunohistochemical analysis of Ki67 was performed. The results showed a remarkable reduction of Ki67 staining in mouse liver organoids treated with 100 µg/mL STDO for 48 h (Figure 2c, Panel 5) compared with LSTDO- and liposome- treated and untreated organoids (Figure 2c, Panels 6, 7 and 8, respectively).

### 2.3. In Vitro Efficacy

**Figure 3:**
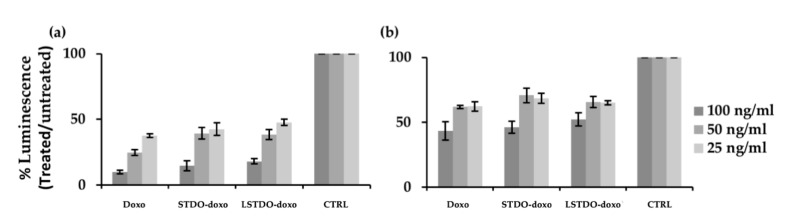
Cytotoxicity of LSTDO-doxo, STDO-doxo and doxo on (**a**) MCF7, (**b**) MDA-MB-231 breast cancer cell lines. Histograms represent the cell viability. Experiments were done in triplicate.

After purification, STDO and LSTDO were loaded with doxorubicin (see Materials and Methods) [17]. The cytotoxic effects of STDO-doxo and LSTDO-doxo were tested on breast (MCF7 and MDA-MB-231) and colon (LoVo) cancer cell lines. The cell viability experiments showed no significant differences among the effects of free doxorubicin, STDO-doxo and LSTDO-doxo (Figure 3 and Supplementary Materials Figure S2). These results are in line with previous publications [5] and are supported by data obtained with liposomal doxorubicin which shown a benefit only in in vivo models [20]. Similarly, our group has previously demonstrated that doxorubicin encapsulated in exosomes, i.e., natural vesicles lined with a bilayer of phospholipids, was able to increase the therapeutic index of this drug only in vivo [21,22].

### 2.4. In Vivo Toxicity

**Figure 4:**
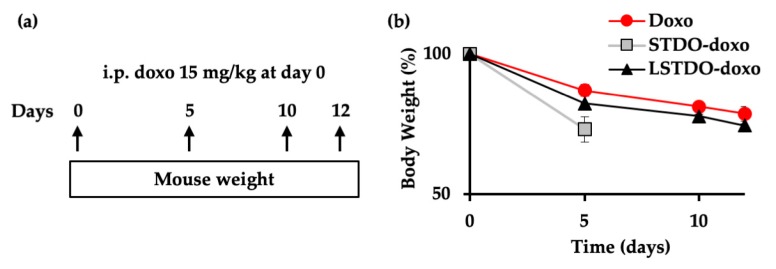
LSTDO-doxo is less toxic than STDO-doxo in vivo. (**a**) Schematic design of the study. Three mice per group were injected intraperitoneally (i.p.) with 15 mg/kg of doxo, STDO-doxo and LSTDO-doxo on day 0 and their body weight was measured at the indicated intervals. (**b**) Mice body weight was followed up for 12 days as an index of illness. Mice treated with LSTDO-doxo w comparable to mice treated with doxo. Mice treated with STDO-doxo had more rapid weight loss and were sacrificed earlier.

To establish the toxic effect of LSTDO-doxo, STDO-doxo and doxorubicin, mice were intraperitoneally (i.p.) injected with a high dose of doxorubicin (15 mg/kg) and the acute toxicity was evaluated (Figure 4a). Body weight was monitored as a quantitative parameter describing animal health, with progressive weight loss indicating deteriorating health. Mice treated with STDO-doxo showed rapid weight loss over five days, after which they were sacrificed. On the other hand, the mice treated with LSTDO-doxo exhibited the same body weight decrease of those treated with free doxorubicin (Figure 4b).

**Figure 5:**
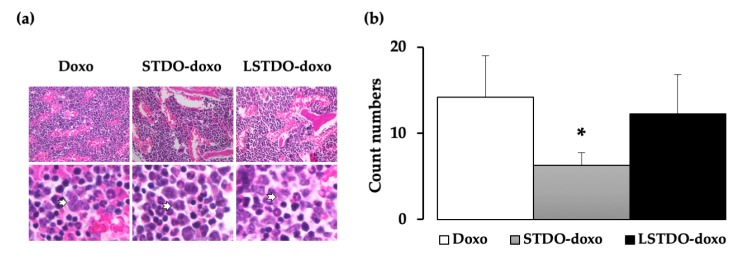
Bone marrow histological analysis of mice treated for acute toxicity. (**a**) The bone marrow was analyzed by histopathology. Representative H&E staining of the bone marrow at 40× magnification (upper panel). Lower panel shows examples of blasts (arrows). (**b**) The number of blast cells was less in STDO-doxo treated mice compared to the other treatments. (* *p*-value < 0.05).

These results showed that LSTDO-doxo was safer than STDO-doxo. To explain the observed toxicity, the tissues of mice were analyzed by histopathology. Among the analyzed tissues (heart, liver, lung, kidney, intestine, spleen and skin; data not shown), we observed a reduced number of blasts in the bone marrow of STDO-doxo treated mice compared to doxo- or LSTDO-doxo-treated mice (Figure 5a,b), which could be a symptom of the observed toxicity.

### 2.5. LSTDO-Doxo is More Effective than Free Doxo In Vivo

**Figure 6:**
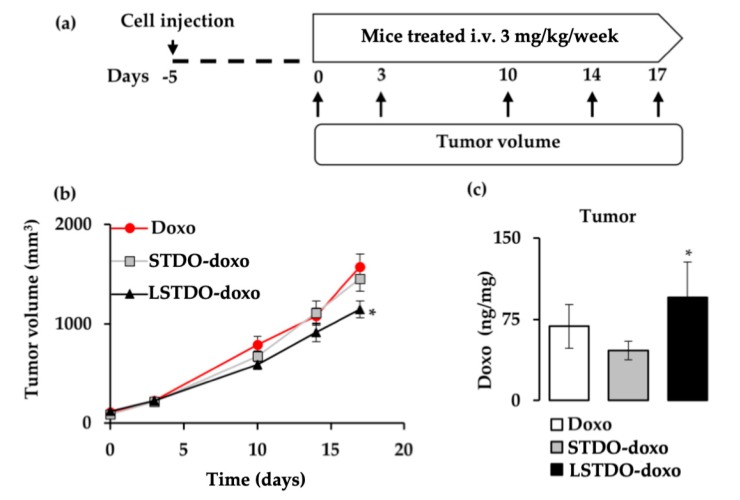
LSTDO-doxo increases the efficacy of doxo in vivo. (**a**) Schematic design of the tumor growth study. Mice were treated three times (3 mg/kg, once a week) and tumor volumes (*n* = 8) were followed up. (**b**) After 14 days, mice treated with LSTDO-doxo had a reduced tumor volume compared with mice treated with doxo and STDO-doxo (* *p*-value < 0.05). (**c**) After 72 h post-doxo injection, mice were sacrificed and tumors (*n* = 8) were collected to quantify the amount of doxo. (ng of doxo/mg of tissue; * *p*-value < 0.05).

To test the antitumor efficacy of LSTDO-doxo, the MDA-MB-231 cell line was orthotopically implanted into the breast of nude mice. After tumors reached >50 mm^3^, mice were treated three times intravenously once per week with 3 mg/kg of doxo, STDO-doxo and LSTDO-doxo. Tumor volumes were followed up (Figure 6a). After 17 days, mice treated with LSTDO-doxo showed a reduced tumor burden compared to mice treated with STDO-doxo or free doxo (27%; *p*-value < 0.05) (Figure 6b). At the end of the experiment, the survival rate of doxo-treated mice was 0%, STDO-doxo was 12.5% and LSTDO-doxo was 33.3%. To support our data, the concentration of doxo was measured in the tumor. As demonstrated in Figure 6c, LSTDO-doxo accumulated in the tumor more than STDO-doxo and free doxo (1.39 fold; *p*-value < 0.05), confirming again that the new DDS presented in this work effectively delivered the drug at the target site, increasing its efficacy.

Although the difference between LSTDO-doxo- and doxo-treated tumors are not impressive, we are proposing an alternative remote loading system based on DNA nanoparticles. Considering that DNA nanostructures have the ability to be functionalized with different chemical groups, we are able to customize the DNA origami accordingly with the drugs to loaded inside the liposomes.

## 3. Discussion

DNA nanotechnology is based on the properties of DNA to form structures through complementary base pair interactions such as robust self-assembly, which allows the design of DNA nanostructures with the required shape, geometry and additional functionalization sites. The modulation of size, shape and net charge of DNA nanostructures has been demonstrated to be important in order to overcome natural cell membrane barriers to deliver naked DNA or siRNA that otherwise would not enter the cells. Another advantageous feature of DNA origami could be the ability to exhibit controlled drug release. However, the in vivo application of DNA nanostructures also presents some limitations. In particular, the major limitation for low-density DNA nanostructures is represented by many circulating enzymes such as DNAses that (especially in tumour cells and the tumor microenvironment) are overrepresented and can degrade it quickly. A dense packaging of DNA helices within a DNA nanostructure is one of the strategies used to increase their stability against DNA-degrading enzymes. The encapsulation of DNA structures under a sheet of biocompatible materials like membranes could be another approach to protect them from recognition and degradation by non-specific immune activation [21,23]. From a therapeutic perspective (comparing the clearance half-life of most anticancer drugs), to obtain good in vivo results, we should develop DNA-based nanostructures with a half-life higher than 30 h to enable their clinical application in drug delivery. For instance, the terminal clearance half-life of doxo, which is among the most widely used chemotherapeutic agent, is around 30 h [24]. DNA nanostructures could be designed in order to create a nanodevice to control the activity of an external enzyme [25]. This additional feature could be exploited to actively release drugs through the modulation of the enzyme activity. This could be a key step for the development of a stealth DNA-based DDS to load drugs inside liposomes only by their interaction with DNA, independently of the pKa of the drugs themselves that could represent an important limitation. Furthermore, the possibility to modify oligonucleotides with functional groups could allow the loading of other drugs. 

The present study highlights the excellent versatility of DNA nanotechnology for the development of innovative DDS with high biocompatibility, improved pharmacokinetic/biodistribution profiles and size/shape-dependent enhanced permeability and retention (EPR) effect [26]. Nonetheless, the cost for the synthesis and purification of this kind of DDS are still elevated, but there are many laboratories working to improve this new technology and some ameliorations were recently reached [25,27,28].

In our previous work, we addressed three key limitations in the bioapplication of DNA origami i.e., their short in vivo stability, non-specific immune system activation and poor cellular uptake, seeding the basis for a new drug remote loading concept. In the present work, we successfully applied the DDS in vivo, demonstrating that the system possesses very strong properties to be a candidate for future therapeutic applications. In particular, LSTDO-doxo was shown to improve the efficacy of doxo on mice bearing breast cancer-derived tumors, reducing tumor burden and decreasing the toxic effect of doxo on bone morrow. Toxicity tests on ex vivo three-dimensional (3D) cultures (mouse liver-derived organoids) that resembled the tissue of origin demonstrated that the LSTDO is less toxic than free DNA origami as evidenced by the caspase 3/7 assay and cytomorphological examination of organoids by H&E staining. Moreover, STDO could interfere with cellular proliferation as confirmed by Ki67 staining reduction compared to untreated and liposome-treated mouse liver organoids.

This work embodies strong and substantial innovations to the previously described DNA-based delivery approaches [7,29] for cancer therapy and enhances the translational prospects of DNA origami-based DDS. There are a lot of limitations for the loading of drugs inside liposomes. In some cases, the poor loading efficiency is a hurdle that prevents the use of liposomal drug formulations [30]. We strongly believe that the technology reported here could be applied to every drug able to interact with DNA, to build other biomimetic drug formulations and to obtain a more efficient and safe multistage system.

This work is a first step towards a new nanotechnology drug delivery era in which DNA nanostructures will play a key role. DNA nanotechnology is taking its first steps into the field of drug delivery and it is a necessary and remarkable step forward that could become the basis for the biomedical and pharmacological application of smart DNA nanodevices to translate this technology into clinics.

## 4. Materials and Methods

### 4.1. Materials and Reagents

MDA-MB-231, MCF7 (human breast cancer) and LoVo (human colorectal cancer) cell lines were grown as indicated by the suppliers. Nude and FVB/N mice were purchased from Harlan Laboratories (Udine, Italy). The experimental procedures were approved by the Italian Ministry of Health and performed in accordance with institutional guidelines. We utilized female mice of 6 weeks of age. Data were reported as mean and standard error of the mean. Oligonucleotides for DNA origami were purchased from IDT Technology (Coralville, IA, USA). M13mp18 single strand plasmids were purchased by Bayou Biolabs, LLC (Metairie, LA, USA).

### 4.2. Self-Assembly of DNA Origami

All DNA origami were assembled on ssDNA M13mp18 as a scaffold at a final concentration of 5 nM. Annealing and assembling of DNA origami were performed in 1× TAE-Mg^2+^ buffer (40 mM Tris-HCl; 20 mM acetic acid; 2 mM EDTA; 12.5 mM magnesium acetate; pH 8.0) in a thermocycler (Eppendorf Mastercycler^®^, Hamburg, Germany) by slowly cooling down from 90 °C to room temperature (RT) in 12 h. STDO was designed with CaDNAno software and assembled with the following protocol: 1× TE-Mg^2+^ buffer (10 mM Tris-HCl, 1 mM EDTA, 16 mM MgCl_2_, pH 8) in a thermocycler by slowly cooling down from 65 °C to 4 °C in 19 h.

### 4.3. DNA Origami Purification

To obtain pure DNA origami structures, eliminating the excess of staple strands and scaffolds, we applied the protocol described by Stahl et al. based on PEG precipitation [18]. The DNA origami mixture was mixed 1:1 (v/v) with precipitation buffer containing 15% PEG 8000 (w/v), 5 mM Tris-HCl, 1 mM EDTA and 505 mM NaCl (all chemicals were purchased from Sigma-Aldrich Merck, Darmstadt, Germany). The solution was mixed by inversion and spun down at 10,000× *g* for 25 min at RT. The supernatant was discarded and the pellet was resuspended in physiological solution.

### 4.4. LSTDO Preparation and Purification

Lipid powders were resuspended in chloroform and dried overnight (ON) with a vacuum pump (EZ-2, SP scientific, Ipswich, UK) to form a lipid cake. The formulation of the lipid cake was: 1,2-distearoyl-sn-glycero-3-phosphocholine, 1,2-dihexadecanoyl-sn-glycero-3-phosphoethanolamine-PEG and cholesterol; 55:5:40). Lipid cake (2 mg) was rehydrated in a STDO solution (800 µg) and extruded ten times through 200 nm and 100 nm Millipore filters (Merck Millipore, Darmstadt, Germany). The excess of DNA origami was eliminated by a cationic resin (IONEX H, C.T.S., City, Italy) interaction. Resin (500 µg) was hydrated in 500 µL of mQ water, washed twice and resuspended in 1× PBS. This solution was mixed 1:1 with the LSTDO solution and incubated at RT in rotation for 1 h. After incubation, the solution was centrifuged at 0.2× *g* for 5 min in order to pellet the resin with free DNA origami and the supernatant containing the purified LSTDO was collected.

### 4.5. Doxorubicin Loading and Release

Doxorubicin-HCl was purchased from Accord (Accord Healthcare, Milan, Italy). LSTDO were incubated with a solution of 2 mg/mL doxorubicin 1:2 for 24 h at RT. The excess of unloaded drugs was eliminated by dialysis (1× PBS, pH 7.4, 15,000 MWCO semi-permeable membrane, 2 h at RT). Empty liposomes were treated with the same loading protocol for LSTDO to be used as a control. Intercalated doxorubicin was dosed by absorbance at 450 nm. The release of doxorubicin from liposomes and LSTDO (50 μg/500 μL) was evaluated with a dialysis membrane with a 15,000 MWCO dipped into 1 L of 1× PBS at pH 7.4 or pH 5.5.

### 4.6. Cell Viability Assay

Cells were seeded in 96-well plates (Becton Dickinson, Franklin Lake, NJ, USA) at a density of 1 × 10^3^ cells/well and incubated for 24 h to allow for the attachment of cells. The cells were incubated with doxo, STDO-doxo and LSTDO-doxo at the same concentrations for 96 h. The cytotoxicity was correlated with the cell viability as evaluated by the CellTiter-Glo^®^ Luminescence Assay (Promega, Madison, WI, USA) with an Infinite200 PRO instrument (Tecan, Männedorf, Switzerland).

### 4.7. Organoid Isolation

Mouse liver organoids were isolated from 8-week-old C57/BL6 mice following the protocol described by Stappenbeck [31]. In brief, a piece of liver was dissected and digested with 2 mg/mL collagenase II for 30 min. The digested tissue was transferred into a clean 15 mL tube with 10 mL of washing medium and centrifugated at 0.5× *g*. The supernatant was discarded and the pellet was resuspended in 10 µL of Cultrex^®^ BME (Trevigen, Gaithersburg, MD, USA) and plated in a 24-well plate. After solidification of the matrix, 500 µL of medium was added to each well.

### 4.8. Toxicity Tests on Mouse Liver Organoids

To test the toxic effect of STDO and LSTDO, mouse liver organoids were plated in 96-well plates and treated with each compound at 100 µg/mL and 10 µg/mL. After 24 h, organoids were analyzed with Caspase-Glo^®^ 3/7 (Promega, Madison, WI, USA). After 48 h, organoids were collected and embedded in paraffin for histopathological analysis.

### 4.9. Histopathology

The organs of mice were collected and fixed in phosphate-buffered 10% formalin, embedded in paraffin, sectioned at a thickness of 3 µm, and stained with hematoxylin and eosin (H&E). The tissues were analyzed with a light microscope using different magnifications. Morphological details were analyzed at 40× objective. Organoids, previously washed with pre-warmed PBS, were embedded using Bio-Agar (Bio-Optica, Milan, Italy) and the blocks were fixed overnight in 10% neutral buffered formalin. Subsequently, samples were processed for paraffin embedding. Sections were used for H&E staining using a Leica ST5020 multi-stainer. Immunohistochemistry (IHC) was performed with the UltraVision LP Detection System HRP DAB kit (Thermo Scientific, Waltham, MA, USA). Heat-induced antigen retrieval was performed using 10 mM citrate buffer, pH 6.0, and the slides were incubated with the following primary antibodies diluted in 0.5% BSA: rabbit anti-E-cadherin (E-cad, Genetex #GTX100443) diluted at 1:500, mouse anti-albumin (ALB (F-8), Santa Cruz #sc-374670) diluted at 1:100, rabbit anti-HNF4α (Abcam #ab181604) diluted at 1:200, rabbit anti-cytokeratin 19 (KRT19, Origene #TA313117) diluted at 1:50, rabbit anti-SOX9 (Millipore #AB5535) diluted at 1:250, rabbit anti-CD133 (Proteintech #18470-1-AP) diluted at 1:200, rabbit anti-OCT4 (Abcam, #18967) diluted at 1:100, and rabbit anti-Ki-67 (Invitrogen, #MA5-14520) diluted at 1:200.

### 4.10. Mouse Xenograft

MDA-MB-231 cells (3 × 10^6^) were mixed with 30% Matrigel HC (BD Bioscience, Franklin Lake, NJ, USA) and implanted orthotopically into 6-week-old female nude mice. When tumors reached a measurable size (>50 mm^3^), mice were treated i.v. with doxo, STDO-doxo and LSTDO-doxo once per week for a total of three treatments. Tumor volumes were measured with a caliper instrument and calculated using the following formula: (length × width^2^)/2.

### 4.11. Biodistribution

Organs of mice were washed with 10 mL of cold PBS/heparin before collection, diluted in 500 µL of 4% PBS/BSA and homogenized with Qiagen Tissue Ruptor for 20 s at power 4 in ice. Samples were then stored at −80 °C. The concentrations of doxo were measured by liquid chromatography tandem mass spectrometry (LC-MS/MS) as described by Bayda et al. [32].

### 4.12. Statistical Analysis

The statistical significance was determined using a two-tailed *t*-test. A *p* value less than 0.05 was considered significant for all comparisons. Bars represent standard errors for tumor volume and body weight. All other bars are standard deviations.

## 5. Conclusions

In this work, we translate in vivo the system previously described in [17]. In particular, before starting the in vivo tests of LSTDO-doxo, we ensure its biocompatibility by performing toxicity tests on mouse liver-derived organoids. The internalization of DNA origami inside the liposomes favored and prevented the toxic effect of free DNA origami. In vivo tests highlighted both an improvement in tumor efficacy and a better drug accumulation in the tumor. We believe that the current work represents a strong and substantial step to translate DNA origami DDS into the clinic.

## Supplementary Materials

The following are available online at https://www.mdpi.com/2072-6694/11/12/1997/s1, Figure S1: Characterization of liposomes, STDO and LSTDO. Figure S2: Efficacy of LSTDO-doxo on LoVo colorectal cancer (CRC) cell lines.
