# Supplementary material for: An Effective Multi-Stage Liposomal DNA Origami Nanosystem for In Vivo Cancer Therapy
Source: Cancers (Basel). 2019 Dec 12;11(12):1997. doi: 10.3390/cancers11121997 (PMC6966502; doi:10.3390/cancers11121997)
Supplement: Supplementary file 1 [file cancers-11-01997-s001.pdf]

cancers

Supplementary Materials: An Effective Multi-Stage Liposomal DNA Origami Nanosystem for in Vivo Cancer Therapy

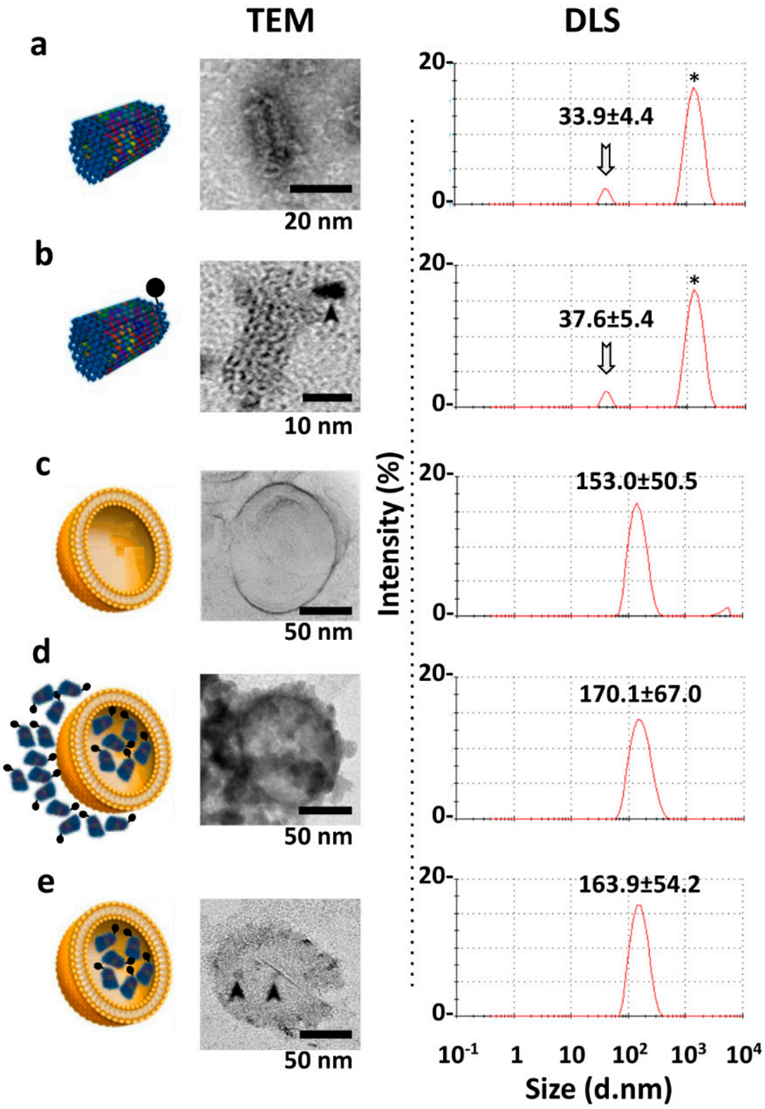

Supplementary Figure S1. Characterization of liposomes, STDO, and LSTDO. (A) STDO was synthesized and characterized by TEM and DLS. (B) To visualize STDO inside liposome, a Cd–Se quantum dot (QD, arrowhead) was conjugated to STDO. (C) Pegylated liposomes were synthesized and (D) loaded with STDO (LSTDO). DLS was able to detect only liposomes (LSTDO). (E) LSTDO was purified by the excess of STDO incubating with a cationic resin that binds unloaded STDO but not liposomes. Arrowheads indicate QD-STDO inside liposomes. Reprinted with permission from Palazzolo et al. [1] Copyright (2019) American Chemical Society.

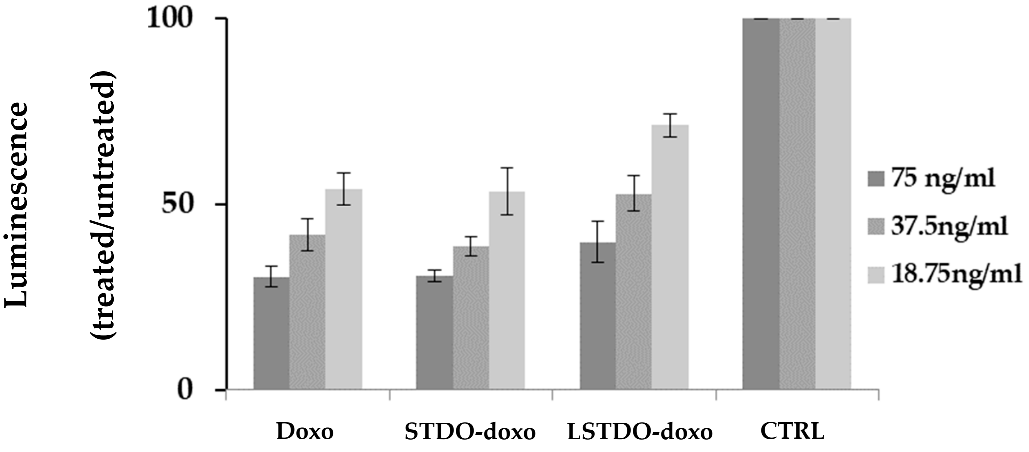

Supplementary Figure S2. Efficacy of LSTDO-doxo on LoVo colorectal cancer (CRC) cell lines. Histograms represent the viability of cells. Experiments were done in triplicates. We used a different tumor cell line to confirm the results obtained on breast cancer cell lines.

Reference

1. Palazzolo, S.; Hadla, M.; Spena, C.R.; Bayda, S.; Kumar, V.; Lo Re, F.; Adeel, M.; Caligiuri, I.; Romano, F.; Corona, G.; et al. Proof-of-Concept Multistage Biomimetic Liposomal DNA Origami Nanosystem for the Remote Loading of Doxorubicin. *ACS Med. Chem. Lett.* **2019**, *10*, 517–521.
